# Renaissance of Traditional Mineral Drugs in Cancer: Advanced Delivery Strategies and Bioengineering Approaches

**DOI:** 10.3390/pharmaceutics18070768

**Published:** 2026-06-23

**Authors:** Aolin Chen, Ping Luo, Jing Cao, Taohong Su, Xinxin Ding, Xinzhi Guo, Wenhao Zhou, Yang Chen, Fang Wang

**Affiliations:** 1 Cancer Research Center, The Jiangxi Province Key Laboratory for Diagnosis, Treatment, and Rehabilitation of Cancer in Chinese Medicine, Jiangxi University of Chinese Medicine, Nanchang 330004, China; chenaolin@jxutcm.edu.cn (A.C.); luoping1018@jxutcm.edu.cn (P.L.); caojing@jxutcm.edu.cn (J.C.); sutaohong@jxutcm.edu.cn (T.S.); dingxinxin1@jxutcm.edu.cn (X.D.); guoxinzhi1@jxutcm.edu.cn (X.G.); zhouwenhao1@jxutcm.edu.cn (W.Z.); 2 State Key Laboratory of Phytochemistry and Natural Medicines, Dalian Institute of Chemical Physics, Chinese Academy of Sciences, Dalian 116023, China

**Keywords:** mineral drugs, cancer, drug delivery, nanomedicine, stimuli-responsive delivery

## Abstract

Traditional mineral drugs represent an underexploited reservoir of natural antitumor agents; however, their clinical translation has historically been hindered by poor bioavailability, non-specific biodistribution, and dose-limiting toxicity. This review comprehensively examines the pharmacological mechanisms and modern formulation strategies driving the renaissance of mineral-based oncology therapeutics. We highlight how mineral drugs exert potent anticancer effects through interconnected pathways, including regulated cell death (e.g., apoptosis, ferroptosis), cell-cycle arrest, and immunomodulation. Crucially, we evaluate recent advances in drug delivery systems, such as liposomes, polymeric nanoparticles, inorganic frameworks, and stimuli-responsive (e.g., pH, redox, enzyme) release systems that successfully overcome traditional pharmacological barriers. These bioengineering strategies not only improve solubility and tumor targeting but also significantly widen the therapeutic window, as evidenced by enhanced tumor suppression and reduced systemic toxicity in preclinical models. Despite this progress, challenges regarding in vivo chemical transformations and tumor heterogeneity remain. Ultimately, we propose a closed-loop “Composition–Mechanism–Delivery” design paradigm to guide future research, facilitating the translation of ethnopharmacological heritage into precision mineral-based therapeutics.

## 1. Introduction

Mineral drugs, derived from natural or synthetic minerals, encompass a diverse range of inorganic substances, as well as certain organic mineral materials such as amber and bitumen [1]. With a history spanning millennia, they constitute an indispensable component of traditional medical systems worldwide. For example, Greco-Roman drugs employed alum as an astringent and hemostatic agent [2]. Within Traditional Chinese Medicine (TCM), mineral drugs hold particular prominence. Classified under the category “minerals and stones”, they are applied according to distinctive therapeutic principles, such as “using toxins to combat toxins”, “softening hardness and dissipating nodules”, and “removing decay to promote tissue regeneration”. Representative examples include cinnabar for calming the mind and gypsum for clearing heat [3].

Their use in managing tumors and pathological accumulations dates back centuries. As early as the Han Dynasty, the Shennong Bencao Jing documented 46 mineral drugs and described their foundational applications, including the use of realgar to treat chills and fever, rodent ulcers, and malignant sores [4]. Subsequent texts, such as the Supplement to the Classic of Famous Physicians, further elaborated on their therapeutic functions, noting that mirabilite could dissolve visceral accumulations, eliminate chronic heat obstruction, expel pathogens, remove stagnant blood, resolve abdominal masses, unblock meridians, and promote urination and menstruation.

In modern cancer, mineral-based drugs and their derivatives have achieved notable progress, with some fundamentally reshaping treatment paradigms for specific cancers [5,6,7,8]. Building on TCM principles, arsenic trioxide (ATO) has been developed into highly effective arsenic-based compounds that have revolutionized the treatment of acute promyelocytic leukemia (APL) [9]. The Realgar-Indigo Naturalis Formula (RIF) has demonstrated efficacy comparable to intravenous ATO and is widely used in China for APL treatment and clinical research [10]. Beyond systemic arsenic therapy, mineral-based biomaterials, such as hydroxyapatite and calcium sulfate, are being developed as local drug delivery carriers or bone repair implants. Their excellent biocompatibility and drug-loading capacity render them promising candidates for postoperative localized therapy and targeted treatment of bone tumors [11]. While robust evidence supports their use in APL, translational research is increasingly exploring their applications in solid tumors, particularly in localized therapy and advanced drug delivery systems [12].

Despite their considerable therapeutic potential, the development of mineral drugs, from traditional remedies to modern oncologic applications, continues to face major pharmaceutical challenges, including poor solubility, non-specific biodistribution, and narrow therapeutic windows [13,14]. Notably, traditional practices provide valuable insights for mitigating toxicity and enhancing efficacy through compatibility-based detoxification and synergistic formulation [15]. Meanwhile, rapid advances in precision drugs, nanoscience, and pharmaceutical chemistry have created unprecedented opportunities for targeted delivery, controlled release, and structural modification of these agents, thereby enhancing their druggability [16,17,18]. Considering these developments, this review summarizes the traditional applications and mechanistic basis of mineral drugs, critically examines their toxicity profiles, and highlights innovative strategies reshaping their development, with the goal of providing a strategic perspective on their clinical revitalization.

## 2. Applications of Mineral Drugs in Cancer Treatment

In cancers, mineral drugs are utilized through two primary and complementary modalities: direct anticancer activity and adjunctive supportive care. One modality involves agents with intrinsic anticancer effects. ATO and realgar, for instance, are prominent agents that demonstrate efficacy against a range of malignancies, including both hematologic cancers and solid tumors [19]. The second modality encompasses minerals that provide critical perioperative support. Magnesium sulfate (Epsom salt) is employed as an osmotic agent to reduce postoperative edema, while gypsum (calcium sulfate dihydrate) is used to promote wound healing and reduce the risk of postoperative infection [20]. Herein, we summarize key mineral drugs, their active components, target indications, and current research status (Table 1).

### 2.1. Anticancer Effects

Multiple mineral-based agents demonstrate clinical potential in cancer therapy through direct antitumor effects. Arsenic compounds, for instance, are well established in the treatment of hematologic malignancies, particularly APL [42]. Preclinical studies have shown that ATO inhibits a variety of tumor types, including prostate cancer [30,31], medulloblastoma [42], hepatocellular carcinoma [43,44], multiple myeloma [38,45], and B-cell acute lymphoblastic leukemia [39]. Clinically, the combination of ATO and all-trans retinoic acid (ATRA) achieves complete remission rates exceeding 90% and 5-year survival rates over 85% in patients with APL, thereby redefining the standard of care for this disease [46].

Historically, mercury compounds were employed both topically and orally in ancient China to manage skin diseases, venereal infections, ulcers, and tumor-like masses [47]. Prior to the nineteenth century, mercuric chloride, mercurous salts, and cinnabar were commonly used as disinfectants and caustic agents to debride necrotic tissue and promote wound re-epithelialization [48]. Selenium, an essential trace element, supports immune function when consumed at appropriate levels and may exert protective effects on normal tissues [49]. Epidemiological studies suggest that high dietary selenium intake is associated with a reduced risk of colorectal cancer (CRC) recurrence [50].

Despite the remarkable success of mineral-based therapeutics in hematologic malignancies such as APL, their application in solid tumors remains at an early stage of exploration [51]. Unlike APL, where arsenic compounds target a defined molecular driver validated by rigorous clinical trials [52], most evidence for solid tumors is derived from preclinical models, including in vitro studies and xenograft experiments [53]. This gap does not diminish the potential of mineral agents; rather, it highlights an exciting frontier for mechanistic discovery and advanced formulation design. Accordingly, the antitumor activities observed in these preclinical settings should be viewed as promising pharmacological leads awaiting clinical validation, rather than established therapeutic effects.

### 2.2. Alleviation of Cancer-Related Complications

Mineral drugs also play a significant role in alleviating symptoms and improving quality of life by addressing specific cancer-related complications, including fluid imbalance and impaired wound healing [54]. Malignant pleural effusion (MPE), for example, is a common complication of lung and breast cancers that often causes dyspnea and chest discomfort [55]. Conventional treatments, such as intrapleural chemotherapy combined with diuretics, provide limited benefit and may induce adverse effects, including electrolyte disturbances, fever, nausea, vomiting, and bone marrow suppression [56]. Mirabilite, recognized for its laxative, anti-inflammatory, and decongestant properties, is widely used in China in combination with rhubarb and intrapleural cisplatin to manage this condition [54].

For gastrointestinal complications, warm magnesium sulfate compresses can accelerate recovery in cancer patients with paralytic ileus by promoting earlier return of bowel function, reducing abdominal discomfort, and decreasing the need for nasogastric decompression [57,58,59]. Topical magnesium sulfate is also beneficial for managing adhesive intestinal obstruction. In supportive care applications, case studies have reported that topically applied magnesium sulfate alleviates refractory scrotal edema, and magnesium-based wet dressings are generally well tolerated [60]. However, high-quality clinical evidence supporting the combined use of Coptis ointment with magnesium sulfate for tumor-related hypoproteinemia-associated scrotal edema remains lacking [61]. Furthermore, borax glycerin has been shown to be effective in managing oral fungal infections by alleviating symptoms, preserving mucosal integrity, and reducing the risk of secondary infection, while maintaining a favorable safety profile in cancer patients [62].

### 2.3. Synergistic Effects with Other Anticancer Therapies

Mineral agents can further enhance therapeutic outcomes through synergistic interactions with other anticancer treatments. A clinical study in patients with chemotherapy-induced oral mucositis found that adding Kangfuxin solution and recombinant human granulocyte colony-stimulating factor (rhG-CSF) to a standard compound borax rinse significantly shortened the time and reduced the incidence and recurrence of mucositis compared with the borax rinse alone, highlighting the clinical value of the combined regimen [63]. Selenium supplementation has also been shown to reduce radiation-induced oxidative stress and improve hepatic and renal function [24]. Similarly, borax and boric acid have been reported to mitigate cisplatin-induced inflammation, oxidative stress, and endoplasmic reticulum stress [23,64,65]. Co-administration of these agents also reduces renal apoptotic damage, demonstrating a significant protective effect [64].

Modern cancer care increasingly incorporates the TCM principle of integrating external applications with internal therapies, offering distinct advantages in reducing postoperative complications and promoting recovery. In gynecologic malignancies, postoperative abdominal distension and gastrointestinal dysfunction are common [22]. Topical application of rhubarb and Epsom salt to the abdomen has been shown to shorten gastrointestinal recovery time, alleviate distension and pain, and demonstrate a favorable safety profile with minimal adverse events [66]. Lymphocele formation frequently occurs following lymph node dissection in gynecologic cancers [67]. Integrating topical rhubarb and mirabilite therapy into standard management not only significantly reduces local pain and prevents infection but also accelerates recovery [25]. Moreover, this adjunctive therapy facilitates cyst resolution, shortens drainage duration, decreases pelvic effusion volume, reduces recurrence rates, and improves overall prognosis in patients [25].

## 3. Representative Antitumor Mineral Drugs

**Figure 1:**
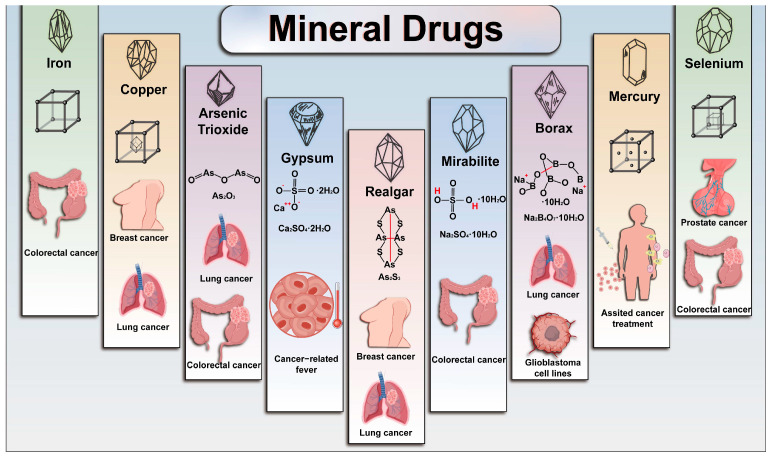
Applications of mineral-based drugs in cancer treatment. This figure summarizes representative mineral drugs, their primary chemical compositions, and their reported associations with various cancers and cancer-related pathological conditions. For each mineral, key chemical structures or crystalline forms are presented alongside the corresponding cancer types or clinical contexts in which potential therapeutic effects have been reported.

The efficacy and safety of mineral drugs are fundamentally determined by their chemical composition, which is further influenced by specific processing methods. Representative agents, such as realgar, ATO, mirabilite, gypsum, and borax, exhibit complex and heterogeneous chemical profiles composed of diverse minerals and trace elements [1]. Beyond their general anticancer and supportive roles, their specific bioactivities, including cytotoxicity, anti-inflammatory, and tissue-repair effects, are directly attributable to these unique compositions [51]. Importantly, most mineral drugs are derived from natural ores and require specialized processing (e.g., crushing, purification, and detoxification) to enhance purity, optimize efficacy, and reduce toxicity (Figure 1).

### 3.1. Realgar

Realgar, an arsenic-containing mineral used in Traditional Chinese Medicine, is composed primarily of arsenic tetrasulfide and arsenic disulfide [68]. Clinically, it is employed for detoxification, antiparasitic therapy, dampness drying, phlegm resolution, antimalarial activity, and antitumor effects, exemplifying the dual pharmacological and toxicological nature of many traditional agents [69]. According to the Encyclopedia of TCM Formulas, realgar appears in 1288 standardized prescriptions and is combined with 939 other medicinal ingredients. It is frequently paired with cinnabar, musk, aconite, and licorice and serves as a core component of classic formulas such as A Gong Niu Huang Wan, Niu Huang Jie Du Pian, and Liu Shen Wan [70]. Within these formulations, realgar functions as an internal “antidote” and enhances heat-clearing, detoxifying, anti-inflammatory, and analgesic effects.

Modern research has substantiated its notable antitumor potential. The RIF, when combined with ATRA, achieves a 100% complete remission rate in non-high-risk APL, with a 2-year disease-free survival of 97% [35]. In pediatric APL, oral RIF combined with consolidation therapy yields an 8-year overall survival rate of 100%, demonstrating efficacy comparable to intravenous ATO while significantly reducing hospitalization duration [71]. Notably, the strongest clinical evidence for realgar-based therapy is currently confined to APL and formulated products such as RIF, rather than realgar administered as a single-agent mineral therapy [72]. Preclinical studies further support the cytotoxicity of realgar against multiple cancer cell lines [21]. It induces dose-dependent apoptosis in NB4 leukemia cells, human CRC cell HCT116, and murine CRC cell CT26 and inhibits tumor growth in vivo [73]. Additionally, it exhibits cytotoxicity against human breast (MCF-7), liver (HepG2), and lung (A549) cancer cells, with nanoformulation enhancing both activity and bioavailability [21]. However, most of this evidence remains preclinical, and further mechanistic and clinical studies are required to establish its therapeutic value [74].

The processing of realgar is crucial for balancing efficacy and safety. Traditional production relies on pyrometallurgical extraction and classical processing techniques [75]. Although the fire-refining method is simple, it is energy-intensive and generates arsenic-containing fumes that pose environmental hazards. The widely used “water-flying method” reduces impurities and toxicity but suffers from low yield and high cost and does not alter the crystalline phase [75]. To address these limitations, modern wet-extraction technologies, including pulverization, slurrying, flotation, chemical purification, and vacuum drying, have been developed, enabling cleaner and more efficient production of pharmaceutical-grade realgar from ore tailings.

### 3.2. Mirabilite

Mirabilite, also known as Glauber’s salt, typically appears as colorless or white crystals. First recorded in Shennong Bencao Jing, it has long been used in TCM to clear heat, promote bowel movements, soften hard masses, resolve nodules, detoxify, and stimulate bile secretion to relieve jaundice [54]. Building on these traditional applications, modern clinical use focuses on treating constipation, inflammation, and hepatobiliary disorders. Upon dissolution in water, mirabilite releases sodium and sulfate ions to form a hypertonic solution, which draws water into the intestinal lumen, stimulates secretion and peristalsis, and promotes defecation while relieving abdominal distension [76]. In clinical practice, classic purgative formulas such as Da Chengqi Tang frequently combine mirabilite with rhubarb to potentiate their laxative effect. Mirabilite also suppresses inflammatory mediator release and has demonstrated therapeutic value in conditions such as enteritis and dermatitis [54]. Therefore, the current clinical utility of mirabilite is primarily observed in supportive or adjunctive care rather than as a direct systemic anticancer agent [25].

Recent studies have highlighted its potential in CRC models. It has been reported that mirabilite significantly reduced adenoma formation and inhibited CRC progression by modulating bile acid metabolism through Lactobacillus enrichment and farnesoid X receptor (FXR) activation [3]. Additionally, oral administration of mirabilite suppresses intestinal gland hyperplasia and carcinogenesis, alters lipid-related biomarkers, and induces metabolic dysregulation in CRC [77,78,79]. Furthermore, mirabilite promotes apoptosis in CRC and reduces carcinoembryonic antigen levels [37].

The processing of mirabilite is designed to moderate its strong purgative effects. According to pharmacopeial procedures, crude mirabilite is decocted with radish, whose properties in TCM theory are believed to temper its harsh nature, followed by filtration, crystallization, and drying to yield a refined product [54]. Industrial processing typically involves dissolving crude ore to obtain a saturated solution, followed by controlled crystallization, filtration, and drying to produce high-purity material.

### 3.3. ATO

ATO is a mineral-derived compound that appears as white to pale-yellow crystals. Historically used to treat conditions such as malignant sores, its modern breakthrough occurred in the 1970s when Tingdong Zhang and colleagues developed an arsenic-based formulation demonstrating remarkable efficacy against APL [46]. This discovery established ATO as a cornerstone of APL therapy [36].

Subsequent clinical trials have confirmed its anticancer efficacy [41]. In APL, combining ATO with ATRA achieves a 100% remission rate and a 98% 2-year event-free survival rate, surpassing outcomes achieved with ATRA plus chemotherapy (98% and 86%, respectively) [80]. The clinical efficacy of ATO in APL is supported by a well-defined disease-specific rationale, as arsenic-based therapy directly targets the PML (Promyelocytic Leukemia)-RARA-driven leukemic program rather than acting solely as a nonspecific cytotoxic agent [81]. Beyond hematologic malignancies, ATO also shows promise in solid tumors. ATO-based whole-cell vaccines induce antitumor immune responses and suppress tumor growth in mouse models of lung carcinoma, fibrosarcomas, and colon carcinoma [82]. In hepatocellular carcinoma, ATO induces mitochondrial damage and activates the cGAS-STING-IFN signaling pathway, promoting CD8^+^ T cell infiltration and inhibiting tumor growth [83]. Furthermore, preclinical studies suggest that combining ATO with olaparib may enhance chemosensitivity through synergistic mechanisms [28]. These findings, while promising, also highlight the mechanistic diversity of ATO beyond its canonical APL target [29].

Traditional preparation of ATO relies on sublimation. Historical texts, including the Kai Bao Ben Cao and Ben Cao Yan Yi from the Song Dynasty, describe processes involving the crushing and roasting of arsenic-containing ores, the collection of the resulting vapors, and their condensation into crystalline products through repeated cycles. Records from the Ming Dynasty further describe co-calcination with alum to produce “alum-calcined arsenic”, a method considered safer but with limited adoption. Modern production typically begins with purified realgar, utilizing calcination and sublimation to generate and condense gaseous As_2_O_3_ [84]. For medicinal use, ATO undergoes additional processing steps, including purification, water grinding, and fine grinding.

### 3.4. Gypsum

Gypsum, a natural mineral composed primarily of calcium sulfate dihydrate (CaSO4·2H_2_O), typically appears as colorless transparent crystals or white masses. In TCM, it is classified as a heat-clearing agent used to reduce internal heat, alleviate restlessness, and relieve thirst. Clinically, it is indicated for conditions such as high fever, lung-heat cough, stomach-fire toothache, and skin ulcers [85]. It is a key component of classic formulations such as Baihu Tang, Maxing Shigan Tang, and Daqinglong Tang.

Modern clinical studies have explored its adjunctive role in cancer. Some gypsum-containing formulations have demonstrated therapeutic benefits in managing cancer-related fever, improving response rates, and shortening symptom duration [86]. In addition, gypsum-based materials have applications in skeletal repair. Calcium sulfate can modulate local inflammation, promote angiogenesis, and support tissue regeneration and fracture healing [87]. In this context, the translational relevance of gypsum lies primarily in its local biomaterial function, postoperative tissue repair, and potential as a drug carrier, rather than in systemic anticancer activity [88].

Processing methods for gypsum have evolved significantly. Historically, gypsum was obtained through natural crystallization or direct ore processing, which resulted in low efficiency and variable product quality [89]. Modern techniques, including hydrothermal and solvent-based extraction, employ elevated temperature and pressure to enhance the release of active components, thereby improving yield and product uniformity while reducing energy consumption [90]. For purification, flotation serves as an effective auxiliary method for removing certain impurities. However, its efficiency is limited when impurities are embedded within the crystal lattice, representing a bottleneck in the production of high-purity gypsum [91].

### 3.5. Other Mineral Drugs with Anticancer or Adjunctive Potential

Several additional mineral drugs have been explored for anticancer or supportive applications, though their clinical evidence remains limited compared with arsenic-based agents. Borax (Na_2_B_4_O_7_·10H_2_O) can induce apoptosis and ferroptosis in hepatocellular carcinoma and glioblastoma cells [27]; however, in clinical oncology, it is primarily used as a local adjunctive intervention for oral fungal infections and mucositis rather than as a systemic anticancer agent [92]. Mercury-containing minerals, most notably cinnabar (HgS), exhibit in vitro cytotoxicity against cancer cells [15] but lack prospective clinical evidence for direct antitumor use [93]. Copper-containing minerals (e.g., cupric oxide) have a long history in TCM for promoting blood circulation and relieving pain. Dysregulated copper metabolism is implicated in tumor development, and copper can induce a distinct form of regulated cell death (cuproptosis) [94,95]. However, most anticancer evidence for copper-containing systems remains mechanistic or preclinical, and elevated copper levels do not by themselves establish clinical efficacy [96]. Collectively, these mineral drugs are best regarded as traditional or supportive agents with modest direct anticancer validation.

In contrast, selenium-containing agents and iron-containing minerals have been developed into modern formulations with distinct functional roles. Selenium supports redox regulation and immune homeostasis; its best-established oncological application lies in chemoprevention, nutritional support, and mitigation of treatment-related toxicity, rather than direct tumor eradication [97]. Selenium nanoparticles (SeNPs) have been engineered to improve bioavailability and targeted delivery [98]. Iron-containing minerals, particularly magnetite (Fe_3_O_4_), have been transformed into superparamagnetic iron oxide nanoparticles (SPIONs), which serve as multifunctional platforms for magnetic targeting, hyperthermia, drug delivery, and ferroptosis-oriented therapy [99].

The preparation of these mineral drugs shares common engineering principles: purification, particle size reduction, and adoption of modern technologies. Borax is obtained by aqueous leaching and crystallization, with green solvent extraction improving purity and reducing environmental impact [100]. Cinnabar is processed through ore selection, grinding, and levigation to remove toxic soluble mercury species [32]. Copper-containing minerals are traditionally processed by ore selection, crushing, grinding, and levigation, sometimes combined with heating or calcination; modern strategies emphasize chemical purification, phase identification, and particle-size control to minimize contamination [33]. Selenium nanoparticles are synthesized via chemical or biological reduction of inorganic selenium salts, often using natural polymers as stabilizers [101]. SPIONs are typically prepared by co-precipitation, thermal decomposition, or hydrothermal synthesis, allowing precise control over size, crystallinity, and surface chemistry—properties that are critical for their performance in nanomedicine [102].

## 4. Mechanism of Mineral Drugs-Mediated Anticancer

**Figure 2:**
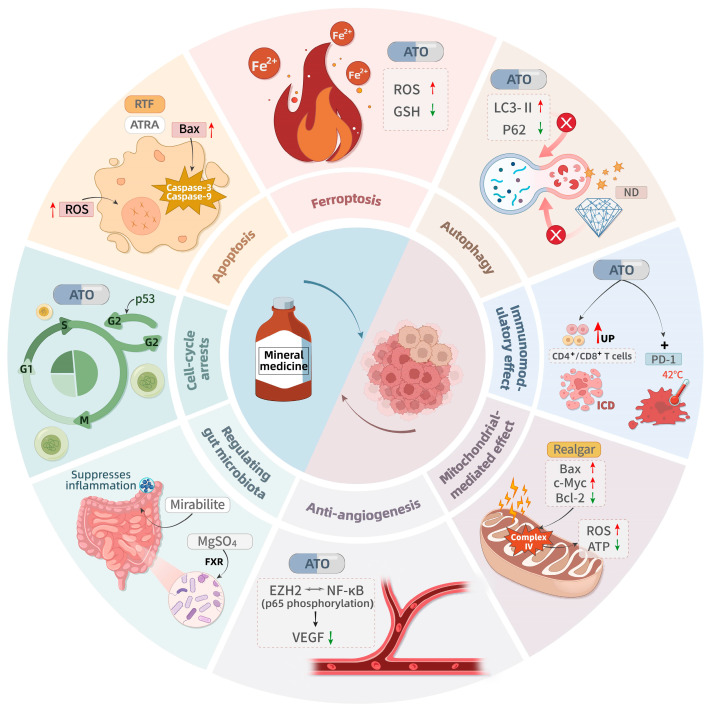
Multifaceted antitumor mechanisms of mineral-based drugs. This figure summarizes representative mineral drugs and their reported mechanisms of action in cancer therapy. The illustrated processes include apoptosis, ferroptosis, autophagy, cell cycle arrest, immunomodulation, mitochondrial dysfunction, anti-angiogenesis, and regulation of the gut microbiota, along with selected molecular events associated with these pathways.

Mineral drugs exert their antitumor effects through a coordinated interplay of direct cytotoxic mechanisms and systemic regulatory modulation (Figure 2). Direct actions include the induction of tumor cell apoptosis, cell cycle arrest, ferroptosis, and inhibition of angiogenesis. Concurrently, these agents modulate broader physiological processes by enhancing antitumor immunity and regulating the gut microbiota, thereby establishing an integrated, host-oriented defense against cancer progression.

### 4.1. Induction of Apoptosis and Cell Cycle Arrest

Tumor development is fundamentally associated with dysregulated cell-cycle control, which drives uncontrolled cellular proliferation [103,104,105,106]. Accordingly, induction of cell-cycle arrest represents a key therapeutic strategy for limiting tumor growth. Mineral drugs, particularly arsenic-based compounds, exert multi-target regulatory effects on the cell cycle and are potent inducers of both cell cycle arrest and apoptosis [107]. Accumulating evidence demonstrates that arsenic-based agents disrupt tumor cell cycle progression through defined molecular mechanisms [108]. For example, Zhang et al. reported that RIF combined with ATRA induces differentiation and apoptosis in NB4 leukemia cells [109]. ATO, in combination with vinorelbine or docetaxel, decreases cell viability and reduces the proportion of SK-N-SH neuroblastoma cells in the G2/M phase, indicating that enhanced cytotoxicity is mediated through G2/M arrest [110]. Wang et al. further demonstrated that in p53-mutant small-cell lung cancer cells, ATO binds to MDM2 (Mouse Double Minute 2 homolog), promoting ubiquitin-mediated degradation of mutant p53 and inducing p53-dependent G2 arrest [111].

Concurrently, arsenic compounds activate intrinsic apoptotic pathways. They modulate the balance of Bcl-2 family proteins (e.g., upregulating Bax) and trigger the activation of caspase-9 and caspase-3 [112]. Collectively, these mechanisms disrupt both the cell cycle and survival signaling in tumor cells.

### 4.2. Induction of Ferroptosis and Modulation of Autophagy

Resistance to ferroptosis is closely linked to tumorigenesis and cancer progression, making its induction a promising therapeutic strategy [113]. Mineral drugs, owing to their unique metal–ion compositions and redox activity, show significant potential to trigger ferroptosis in tumor cells [114]. ATO serves as a representative ferroptosis inducer. ATO-based nanoparticles deplete or inhibit the synthesis of glutathione (GSH) [113]. Severe GSH depletion limits substrate availability for glutathione peroxidase 4 (GPX4), impairing its ability to detoxify lipid peroxides. Consequently, the accumulation of lipid peroxides in cell membranes leads to ferroptotic cell death [115].

Simultaneously, mineral drugs modulate autophagic activity, which interacts dynamically with ferroptosis. Wu et al. reported that ATO increases LC3-II and decreases p62 expression in osteosarcoma cells, indicating enhanced autophagic flux [116]. In cervical cancer cells, combining cyclosporine A with ATO further elevates LC3-II, suggesting synergistic induction of autophagy [117]. Autophagy plays a context-dependent role in ferroptosis; it can mitigate cellular damage by removing toxic components, yet excessive autophagy may accelerate ferroptotic cell death [118]. Furthermore, Cui et al. demonstrated that nanodiamonds (NDs) inhibit autophagy by blocking autophagosome–lysosome fusion. When combined with ATO, NDs significantly enhance the suppression of proliferation and metastasis in liver cancer [118], highlighting how modulation of autophagy can potentiate the anticancer efficacy of mineral-based agents. From a delivery perspective, ferroptosis-oriented nanocarriers can enhance iron-dependent lipid peroxidation by increasing intracellular iron availability, promoting metal ion release in lysosomes or the acidic tumor microenvironment, facilitating Fenton or Fenton-like reactions, and compromising the GSH/GPX4 antioxidant defense system [119].

### 4.3. Mitochondrial-Mediated Effects

Mitochondria are key targets for the cytotoxic actions of multiple mineral drugs, which induce apoptosis primarily by disrupting mitochondrial integrity and function. Realgar induces apoptosis by triggering mitochondrial membrane potential collapse and cytochrome c release [120,121,122,123]. In chronic lymphocytic leukemia MEC-1 cells, it upregulates pro-apoptotic Bax and downregulates anti-apoptotic Bcl-2, thereby activating the mitochondrial apoptotic pathway and suppressing proliferation [120]. Additionally, realgar disrupts mitochondrial respiratory chain complex IV, reducing ATP production while increasing Reactive Oxygen Species (ROS), leading to oxidative stress and mitochondrial DNA damage [124]. ATO similarly induces membrane potential loss and mitochondrial permeability transition pore (mPTP) opening [125]. Sustained mPTP opening disrupts mitochondrial homeostasis, leading to swelling, outer membrane permeabilization, and release of cytochrome c into the cytosol [126].

Mercury ions (Hg^2+^), derived from cinnabar, inhibit respiratory-chain complex I (NADH dehydrogenase), membrane potential dissipation, and apoptosis [124]. However, chronic exposure may lead to mercury accumulation in organs such as the kidneys and brain, potentially causing neurotoxicity and mitochondrial dysfunction [127]. Beyond conventional mineral agents, modern approaches aim to achieve precise tumor targeting with minimal off-target toxicity. A representative example is boron neutron capture therapy, which exploits nuclear reactions between boron-10 and thermal neutrons to generate high-energy particles with extremely short path lengths, thereby inducing localized damage to DNA and mitochondria and enabling highly selective tumor cell killing [128].

### 4.4. Anti-Angiogenesis

Angiogenesis is essential for tumor progression, supplying nutrients and oxygen while facilitating waste removal. Therefore, inhibition of angiogenesis represents a key therapeutic strategy [129]. ATO suppresses neovascularization through multiple pathways. In triple-negative breast cancer, it disrupts the Enhancer of Zeste Homolog 2(EZH2)-p65 interaction, enhances p65 phosphorylation, and reduces vascular endothelial growth factor secretion, thereby inhibiting angiogenesis [130]. Furthermore, Sun et al. demonstrated that ATO upregulates forkhead box O3a (FoxO3a), significantly impairing angiogenic activity. Restoration of angiogenesis following FoxO3a knockout confirms its role as a key mediator of ATO’s anti-angiogenic effect [131].

Realgar-containing serum (RTS) also exhibits potent anti-angiogenic activity. In zebrafish and chicken embryo chorioallantoic membrane (CAM) models, RTS markedly inhibits angiogenesis in vivo [132]. In an H22 murine hepatoma allograft model established in Kunming (KM) mice, RTS reduced tumor volume by more than 50%. Its anti-angiogenic activity was further supported by CD31 immunohistochemistry and alginate-encapsulated tumor cell assays [132]. Supporting evidence from a CRC mouse model shows that engineered stromal cells expressing soluble Flt-1nearly abolished tumor neovascularization, in contrast to the abundant vasculature observed in control groups [133]. Collectively, these findings underscore the therapeutic potential of mineral-based agents in targeting tumor angiogenesis.

### 4.5. Immunomodulatory Effects

Mineral drugs can reshape the tumor microenvironment and enhance antitumor immunity through diverse immunomodulatory mechanisms. ATO and its derivatives act as potent immunomodulators, primarily by inducing immunogenic cell death (ICD) [134]. In mouse tumor models, ATO-induced ICD promotes effector molecule production by CD8^+^ and CD4^+^ T cells [82]. Genetic knockout studies revealed that this preventive immunity depends critically on ferroptosis-related and necroptosis-related genes, while being only partially affected by the disruption of apoptosis or autophagy pathways [82]. Moreover, combining ATO-based vaccines with PD-1 blockade significantly suppressed tumor growth, suggesting that ATO can convert immunologically “cold” tumors into “hot” tumors [82]. Beyond adaptive immunity, ATO also enhances innate immune responses by increasing natural killer cell cytotoxicity [135]. Other mineral drugs contribute to immune regulation through distinct pathways. Mirabilite enhances macrophage phagocytosis and cytokine secretion [3], and clinical observations suggest that it may reduce the serum level of pro-inflammatory cytokines such as IL-6 (Interleukin-6), IFN-γ (Interferon-γ), and TNF-α (Tumor Necrosis Factor-α) in CRC patients, indicating a role in mitigating inflammation-driven tumor progression. Together, these findings highlight the capacity of mineral drugs to engage multiple components of the immune system, thereby offering promising strategies for cancer immunotherapy.

### 4.6. Regulation of the Gut Microbiota

Emerging evidence indicates that mineral drugs exert antitumor effects by modulating the gut microbiota and its associated metabolic networks, thereby influencing local and systemic tumor microenvironments [136]. A representative example is mirabilite. In an *APC^Min/+^* mouse model of CRC, it suppresses tumor growth, improves intestinal epithelial integrity, and reduces adenoma formation and inflammation [3]. Mechanistically, mirabilite acts through the gut microbiota-bile acid metabolic axis. Metabolomic and network pharmacology analyses demonstrate that it significantly alters microbiota composition and function, thereby reshaping bile acid homeostasis [3]. Specifically, treatment modifies the balance of primary and secondary bile acids, leading to activation of the FXR signaling pathway. FXR activation further optimizes bile acid metabolism, suppresses inflammation, and promotes epithelial repair, ultimately establishing a beneficial antitumor feedback loop mediated by microbiota–host interactions [137].

In addition to mirabilite, selenium-containing agents have also been linked to microbiota-mediated regulation in tumor-bearing models [138]. In mice with breast cancer maintained on a high-fat diet, selenium intervention altered gut microbiota composition and diversity, accompanied by changes in microbial functional pathways related to carbohydrate-active enzymes, membrane transporters, virulence factors, and metabolic potential [138]. In CRC-bearing nude mice, different selenium species, including sodium selenite and selenomethionine, were also reported to influence intestinal microbial communities. These findings suggest that selenium-containing agents may affect tumor biology not only through redox and immune regulation but also by remodeling of intestinal microbial metabolism [139].

The gut microbiota may also influence the metabolism, disposition, and toxicity of arsenic-containing mineral drugs, including ATO and realgar-derived preparations [140]. Experimental evidence indicates that antibiotic-treated or germ-free mice excrete less arsenic in feces and accumulate more arsenic in organs than conventionally raised mice, suggesting that the gut microbiome contributes to arsenic elimination and protection against acute arsenic toxicity. Additional studies further indicate that arsenic exposure can perturb gut microbial composition and metabolic function [141]. Therefore, for arsenic-based mineral drugs, regulation of the gut microbiota may be more related to pharmacokinetics and safety than to a clearly established anticancer mechanism [142].

Iron- and copper-containing systems further illustrate the dual implications of mineral–microbiota interactions [143]. Iron availability is an important determinant of colonic microbial ecology, as many pathogenic bacteria possess strong iron acquisition systems and may outcompete protective commensal bacteria under iron-rich conditions [144]. Copper nanoparticles have also been shown to alter intestinal microbial metabolism; compared with copper carbonate, dietary copper nanoparticles suppressed cecal bacterial enzymatic activity and reduced short-chain fatty acid production in rats [145]. These observations suggest that microbiota-related effects of mineral drugs may represent both a therapeutic opportunity and a potential safety concern.

## 5. Toxicity of Mineral Drugs and Detoxification Strategies

**Figure 3:**
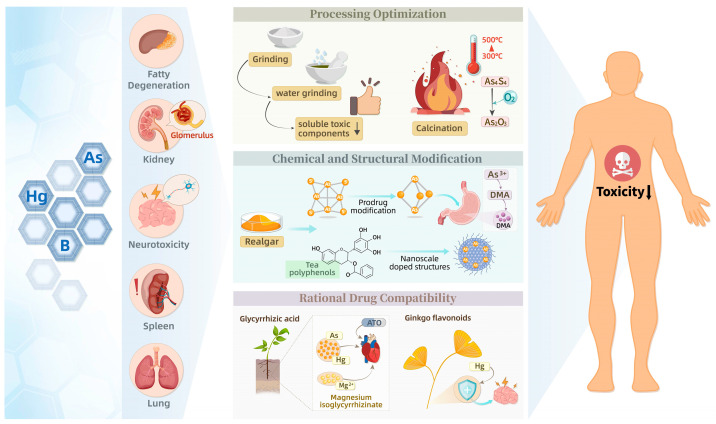
Toxicity of mineral-based drugs and representative detoxification strategies. This figure summarizes the major toxic effects associated with mineral-related elements, including arsenic (As), mercury (Hg), and boron, as well as the corresponding strategies employed to mitigate their toxicity. The illustrated toxicities include fatty degeneration, glomerular injury, neurotoxicity, and spleen and lung damage. Key detoxification approaches comprise processing-induced detoxification, structural or modification-based detoxification, and compatibility-based detoxification.

Although mineral drugs have demonstrated therapeutic efficacy in cancer treatment, their intrinsic chemical properties, especially those of heavy metals and metalloids, combined with improper processing or administration, can lead to significant toxic side effects. Therefore, detoxification strategies are essential to ensure their safe clinical application. Advanced analytical techniques are increasingly employed to elucidate the material basis of toxicity and its metabolic transformations in vivo, thereby providing a robust foundation for safety evaluation and the rational use of mineral drugs (Figure 3).

### 5.1. Toxicity

The therapeutic use of mineral drugs is inherently associated with the potential toxicity of their inorganic constituents, particularly heavy metals and metalloids such as arsenic, mercury, and boron. Therefore, systematic evaluation of their toxicological profiles, particularly with respect to the hepatic, renal, and neurological systems, is essential.

Arsenic toxicity primarily manifests as hepatotoxicity, nephrotoxicity, and neurotoxicity [146]. Chronic exposure leads to bioaccumulation in organs such as the liver and kidneys, resulting in functional impairment. In animal models, arsenic-contaminated diets elevate serum alanine aminotransferase (ALT), aspartate aminotransferase (AST), and urea levels, accompanied by degenerative and inflammatory tissue damage [147]. In the kidneys, arsenic disrupts mitochondrial function in tubular epithelial cells, suppresses antioxidant defenses, and induces pathological changes, including tubular necrosis, glomerular atrophy, and interstitial fibrosis [148]. It also activates inflammatory signaling pathways, such as NF-κB and MAPK (Mitogen-Activated Protein Kinase), thereby increasing pro-inflammatory cytokine expression and exacerbating injury [149]. Neurobehavioral abnormalities have also been reported, particularly during developmental stages.

Mercury is a potent neurotoxin and nephrotoxin. Its toxicity is largely attributed to the irreversible inhibition of critical selenoproteins, such as thioredoxin reductase, thereby disrupting both neurological and renal function [150]. Exposure occurs through inhalation of elemental mercury or ingestion of its inorganic (e.g., cinnabar) and organic (e.g., methylmercury) forms. Inorganic mercury can accumulate in the brain and connective tissues at high doses, whereas organic methylmercury, identified by the World Health Organization (WHO) as a major public health concern, readily crosses the blood–brain barrier, leading to motor and cognitive deficits [151]. Chronic renal accumulation may result in progressive kidney injury and eventual failure. The severe public health impact of mercury is exemplified by Minamata disease, underscoring its classification by the WHO as a priority chemical hazard [152].

In contrast to its traditional applications, boric acid has well-documented systemic toxicity. Dermal exposure, particularly on compromised skin or large surface areas such as the abdomen, can result in significant systemic absorption, although intact skin provides an effective barrier [153]. Oral ingestion is potentially lethal, and animal studies have demonstrated reproductive toxicity, including reduced fertility. Boron is primarily excreted via the kidneys; chronic high-level exposure may lead to pulmonary irritation and genotoxic effects on the reproductive system [153]. Although these studies collectively highlight the major toxicological risks associated with mineral drugs, qualitative descriptions alone are insufficient for comparing their relative safety profiles across different chemical forms and formulations [154]. Herein, we summarize representative acute toxicity indices, organ-specific toxicological endpoints, formulation-dependent safety features, and available safety margin data for various mineral drugs and their formulations (Table 2).

### 5.2. Detoxification Strategies

To ensure the safe clinical application of mineral drugs, multiple detoxification strategies have been developed, primarily focusing on processing optimization, rational drug compatibility, and chemical or structural modification. These approaches aim to reduce the bioavailability of toxic species while preserving or enhancing therapeutic efficacy.

#### 5.2.1. Processing Optimization

Optimization of processing methods represents a foundational detoxification strategy, primarily achieved through traditional techniques such as levigation and calcination. Levigation removes soluble toxic components via repeated aqueous processing at ambient temperature [159]. Metabolomic studies demonstrate that levigated realgar produces fewer toxic hepatic metabolites and exhibits reduced arsenic bioaccessibility in simulated gastrointestinal models, indicating a shift toward less absorbable arsenic species. Notably, studies employing gastrointestinal extraction and diffusive gradients in thin films techniques have shown that arsenic in realgar-containing drugs exhibits low bioaccessible fractions, suggesting that processing alters arsenic dissolution behavior and reduces toxicity potential [160]. Calcination, on the other hand, alters the chemical form of arsenic with, for instance, oxidation from As_4_S_4_ to As_2_O_3_. Such thermal transformations influence arsenic speciation and solid-state characteristics, which are critical determinants of bioavailability. Comparative mineralogical studies have demonstrated that variations in arsenic speciation and host mineral phases result in markedly different bioaccessible fractions under identical conditions [161]. These findings support the concept that processing-induced speciation changes can modulate arsenic exposure. Importantly, the resulting toxicity profile is highly temperature-dependent, highlighting the necessity for precise control of processing conditions.

#### 5.2.2. Rational Drug Compatibility

Rational drug compatibility provides an effective strategy for mitigating toxicity through synergistic pharmacological interactions. This approach includes the use of specific herbal antagonists, such as glycyrrhizic acid and magnesium isoglycyrrhizinate, which can bind arsenic and ameliorate ATO-induced cardiotoxicity via antioxidant, anti-inflammatory, and anti-apoptotic mechanisms [162]. Similarly, flavonoids derived from medicinal plants such as licorice and ginkgo exhibit protective effects by chelating heavy metals, thereby reducing their bioavailability and associated oxidative damage [163]. This principle is exemplified in established formulations such as the Compound Huangdai Tablet (CHDT), which integrates realgar with other botanical components. Clinical evidence indicates that CHDT combined with ATRA achieved a remission rate comparable to ATO monotherapy in APL, while exhibiting reduced toxicity [164]. Furthermore, nanotechnology -based strategies have enabled innovative compatibility approaches. For instance, the encapsulation of realgar with tea polyphenols into nanoparticles [e.g., (-)-Epigallocatechin-3-Gallate Encapsulated Realgar Nanoparticles (EGCG-RNPs)] enhances antitumor activity while minimizing systemic arsenic exposure [165].

#### 5.2.3. Chemical and Structural Modification

Chemical and structural modification represents an additional strategy to enhance safety by rationally altering the physicochemical properties of mineral agents. Chemical derivatization, such as converting inorganic arsenic into organic derivatives (e.g., dimethylarsinic acid), can reduce acute toxicity, improve bioavailability, and enhance selective cytotoxicity toward cancer cells [166]. Structural modification also plays a critical role. For instance, the low-solubility sulfide lattice of realgar slows arsenic release, resulting in reduced hepatotoxicity and neurotoxicity compared to soluble ATO [167]. Adjusting the oxidation state of metal components may further reduce toxicity by decreasing their affinity for biological macromolecules and facilitating excretion [168]. In addition, the design of composite materials, such as hybrid systems incorporating realgar with tea polyphenols or nanoscale doped structures, enables precise control over crystal structure and surface chemistry. These modifications regulate dissolution kinetics and biological interactions, thereby improving both therapeutic efficacy and safety profiles [169]. While these detoxification strategies illustrate how processing, compatibility, and structural modification can reduce mineral-related toxicity, their translational value cannot be fully evaluated based on mechanistic descriptions alone [154]. To address the need for more concrete and quantitative comparisons, representative mineral-based formulations with reported efficacy, pharmacokinetic, biodistribution, or release-related parameters are summarized (Table 3).

## 6. Innovative Delivery and Development of Antitumor Mineral Drugs

**Figure 4:**
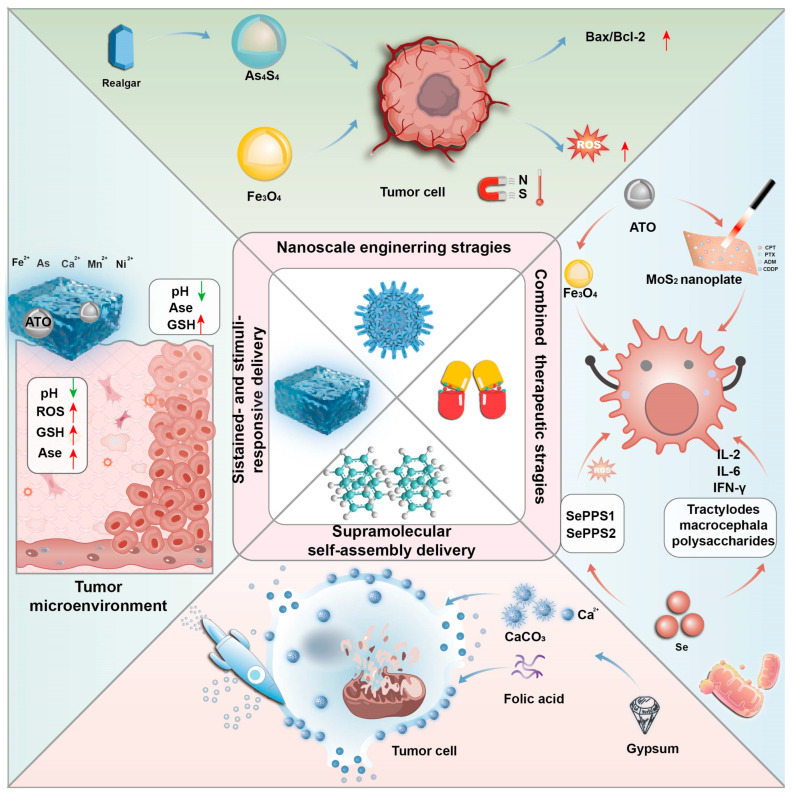
Innovative nanoscale development strategies for mineral-based drugs in cancer treatment. This figure summarizes representative nanoscale engineering strategies for mineral drugs and their applications in cancer treatment. The illustrated approaches include tumor microenvironment-responsive release, sustained-release delivery, supramolecular self-assembly, and combination therapeutic strategies, along with their roles in enhancing tumor targeting, intracellular delivery, immune modulation, and antitumor efficacy.

Beyond intrinsic toxicity, the clinical translation of mineral drugs is often limited by poor aqueous solubility and low target specificity. These limitations contribute to suboptimal therapeutic efficacy, increased systemic exposure, and a narrow therapeutic window. Recent advances in nanotechnology and drug delivery systems provide transformative solutions to these challenges [177]. Innovative strategies, including stimuli-responsive controlled release, supramolecular self-assembly, and synergistic co-delivery platforms, enable the precise encapsulation and functional engineering of mineral agents into advanced nanomedicines. These technologies enhance drug stability and bioavailability, allow spatiotemporal control over drug release, improve targeting precision, and introduce intelligent regulatory functions (Figure 4). Importantly, these delivery systems are not merely formulation improvements but also reinforce specific anticancer mechanisms by enhancing tumor-selective accumulation, enabling controlled metal ion release, modulating redox homeostasis, inducing organelle stress, and promoting combination-based therapeutic synergy [178].

### 6.1. Nanoscale Engineering Strategies

Mineral drugs are rich in inorganic and metallic components, rendering them inherently suitable for nanoengineering. Conversion into nanoparticles increases surface area and enables precise modulation of surface properties, thereby enhancing tumor penetration, retention, and therapeutic efficacy [179]. For example, transforming the active component of realgar (As_4_S_4_) into nanoparticles via high-energy ball milling or related nanofabrication techniques significantly enhances surface area, dissolution rate, and bioavailability. These nanoparticles exhibit improved cellular uptake, inhibit tumor cell proliferation, induce apoptosis, and regulate the Bax/Bcl-2 ratio, leading to antitumor activity [180].

Certain mineral-derived nanoparticles also exploit intrinsic physicochemical properties for novel therapeutic modalities. Ferroferric oxide (Fe_3_O_4_) nanoparticles, derived from magnetite, generate hyperthermia under an alternating magnetic field, enabling tumor ablation. This process further activates cellular stress pathways, releases iron ions, induces ROS generation, and triggers ferroptosis, resulting in synergistic antitumor effects [181]. Mechanistically, Fe_3_O_4_-based nanoplatforms are particularly relevant to ferroptosis because they act as intracellular iron reservoirs [182]. Following cellular internalization, acidic endosomal or lysosomal conditions facilitate iron release and redox cycling, thereby promoting hydroxyl radical generation, lipid peroxide accumulation, and iron-dependent tumor cell death [119]. The functionality can be further enhanced through rational design. Drug loading, via adsorption, conjugation, or encapsulation, improves payload delivery efficiency [183], while surface modification with polyethylene glycol (PEG) and targeting ligands facilitates receptor-mediated endocytosis and enhances therapeutic selectivity and imaging capability [184]. Additionally, integration with ROS-responsive catalytic components enables the construction of “self-amplifying” systems that elevate hydrogen peroxide levels and intensifies Fenton reactions, thereby enhancing photothermal, photodynamic, and chemotherapeutic outcomes [185]. Although these nanoscale engineering strategies demonstrate the potential of mineral-based formulations to improve solubility, tumor penetration, and therapeutic efficacy, descriptive examples alone are insufficient to evaluate their formulation-dependent advantages [186]. To provide a more concrete and quantitative comparison, representative mineral-based delivery systems with reported efficacy, pharmacokinetic, biodistribution, or release-related parameters are summarized (Table 4). These examples include arsenic-based liposomes and polymeric microspheres, nano-realgar hydrogels, iron-based nanocomposites, copper oxide-based drug carriers, and selenium-containing nanoplatforms.

### 6.2. Sustained- and Stimuli-Responsive Delivery

The distinctive biochemical characteristics of the tumor microenvironment, including acidic pH, elevated ROS and glutathione levels, and overexpression of enzymes such as matrix metalloproteinases and hyaluronidase, provide key triggers for designing responsive delivery systems [190,191,192,193]. Metal ions inherent to mineral drugs (e.g., As, Fe^2+^, Mn^2+^, Ca^2+^) can coordinate with functional ligands to form nanostructures capable of releasing therapeutic payloads in response to specific TME (tumor microenvironment)-specific stimuli or external triggers. Mechanistically, stimuli-responsive delivery systems convert tumor-associated biochemical abnormalities into release signals. By restricting mineral ion release or drug activation to tumor tissues and intracellular compartments, these systems enhance local cytotoxic stress while minimizing premature systemic exposure [194].

pH-responsive systems are widely investigated. ATO can be formulated into nanosystems that release drugs under acidic conditions or upon GSH depletion, thereby enhancing apoptosis in APL, hepatocellular carcinoma, and lung cancer [186]. Mn^2+^-based platforms exemplify theragnostic systems, remaining stable at physiological pH but dissociating within the acidic, H_2_O_2_-rich TME. Released Mn^2+^ ions function as activatable Magnetic Resonance Imaging (MRI) contrast agents while catalyzing H_2_O_2_ decomposition, depleting GSH, alleviating hypoxia, and enhancing therapeutic efficacy [195]. Innovative systems such as LP@MnAs, which encapsulate Mn^2+^-arsenate complexes, enable stable circulation and sustained, TME-triggered release, thereby improving tumor accumulation and cytotoxicity [196]. Similarly, realgar quantum dots embedded in a dextran-hyaluronic acid hydrogel form a pH-responsive platform that enhances bioavailability and acts as a sustained ROS generator, ultimately increasing tumor radiosensitivity [172].

Enzyme-responsive strategies provide an additional layer of specificity. For example, GEM-MNP-pHLIP (G-PON Encapsulation Mode-Magnetic Nanoparticles-PH-Low Insertion Peptide) nanoparticles incorporate an Fe_3_O_4_ core for MRI and magnetic targeting, along with a peptide linker cleavable by matrix metalloproteinase-2 (MMP-2), enabling enzyme-triggered drug release [197]. When loaded with gemcitabine, this system demonstrates synergistic cytotoxicity and antiproliferative effects, illustrating the potential of multi-stimuli-responsive mineral-based delivery systems [198].

### 6.3. Supramolecular Self-Assembly Delivery

Supramolecular self-assembly, driven by noncovalent interactions such as hydrogen bonding and van der Waals forces, represents a biomimetic strategy for advanced delivery [199]. This concept is partly inspired by traditional practices, such as the co-decoction of gypsum with licorice, which forms supramolecular aggregates that enhance dissolution and absorption of active components [200]. Modern research has extended this principle to engineered systems. An “intracellular Ca^2+^ nanogenerator”, constructed from calcium carbonate and calcium peroxide, induces calcium overload following cellular uptake, leading to mitochondrial dysfunction and programmed cell death [201].

Further refinements enable targeted delivery. Systems functionalized with folate and bisphosphonate ligands achieve selective tumor recognition and localized biomineralization [202]. More advanced platforms incorporating calcium carbonate nanoparticles, folic acid, and supramolecular peptides undergo intracellular self-assembly in spermine-rich tumor cells, forming micrometer-scale aggregates that prolong retention, induce calcium overload, disrupt mitochondrial integrity, and trigger apoptosis [203]. Thus, supramolecular delivery provides a clear example of the “Composition–Mechanism–Delivery” principle, in which calcium-based composition, mitochondrial injury, and intracellular self-assembly are integrated into a unified therapeutic design that directly links delivery behavior to anticancer mechanisms [204].

### 6.4. Combined Therapeutic Strategies

Given the complexity and adaptability of tumors, monotherapies often exhibit limited efficacy. Consequently, multimodal combination strategies have emerged as a key direction in cancer [205]. Mineral-based agents serve as versatile components in such regimens, demonstrating synergy with immunotherapy, radiotherapy, and chemotherapy. For example, combining ATO with Fe_3_O_4_ nanoparticles or MoS_2_ nanosheets can remodel the tumor microenvironment and promote ferroptosis. When further combined with anti-PD-L1 checkpoint blockade, this strategy significantly enhances antitumor efficacy [206]. This strategy exemplifies mechanism-oriented co-delivery: Fe_3_O_4_ nanoparticles serve as an iron-related redox source for ferroptosis, ATO induces oxidative stress and cell death signaling, MoS_2_ nanosheets enhance photothermal stress and remodel the tumor microenvironment, and anti-PD-L1 blockade amplifies antitumor immunity [29]. MoS_2_ nanosheets also enable synergistic photothermal–chemotherapy effects due to their high surface area and photothermal conversion efficiency [207].

Another promising approach involves combining mineral elements with bioactive natural products. Selenium-modified polysaccharides exhibit enhanced antitumor activity by increasing ROS production, disrupting mitochondrial function, and inducing apoptosis [208]. Additionally, metal–flavonoid complexes enable sustained ion release and synergistic biological effects, further expanding therapeutic potential [209]. These findings highlight the broad potential of mineral-based agents in rational combination therapies. By establishing synergistic interactions with both modern treatment modalities and refined natural products, they offer a pathway toward more integrated and effective therapeutic strategies in cancer.

## 7. Conclusions, Limitations, and Future Perspectives

Mineral drugs constitute a unique and underexplored class of therapeutic agents for cancer treatment [210]. Unlike conventional small-molecule drugs, their biological activities arise from complex physicochemical properties, including metal speciation, crystalline structure, redox behavior, and dynamic interactions with biological systems [211]. As summarized throughout this review, mineral drugs can regulate multiple hallmarks of cancer through diverse mechanisms, including apoptosis induction, ferroptosis activation, redox modulation, immune regulation, metabolic reprogramming, and tumor microenvironment remodeling [212]. These characteristics provide opportunities for the development of novel anticancer strategies that complement existing therapeutic modalities.

Despite these promising advances, several major challenges continue to hinder clinical translation [213]. First, the pharmacokinetic behavior, biodistribution, metabolic transformation, and long-term safety profiles of many mineral agents remain insufficiently characterized [214]. Because their biological activities are highly dependent on chemical speciation, particle size, solubility, crystalline phase, and in vivo transformation, therapeutic efficacy and toxicity cannot be reliably predicted from composition alone [215]. Second, mineral drugs frequently exhibit context-dependent biological activities. Variations in tumor genotype, redox status, metabolic state, and microenvironmental conditions can profoundly influence their mechanisms of action and therapeutic responses [216]. Third, the abnormal architecture and heterogeneity of solid tumors often limit drug penetration and produce highly variable intratumoral exposure, thereby reducing translational predictability. Collectively, these factors contribute to the complexity and uncertainty associated with the clinical development of mineral-based therapeutics [217].

Recent advances in nanotechnology, biomaterials, and precision delivery systems provide promising solutions to many of these challenges [218]. Stimuli-responsive nanocarriers, biomimetic delivery platforms, and rationally engineered nanostructures have demonstrated the ability to improve tumor targeting, regulate metal ion release, reduce off-target toxicity, and enhance therapeutic efficacy [219]. More importantly, modern delivery systems are increasingly serving not only as pharmacokinetic optimization tools but also as active regulators of therapeutic mechanisms, enabling precise control of redox signaling, ferroptosis induction, organelle stress, and immune activation within tumor tissues [190].

Looking forward, future research should move beyond proof-of-concept efficacy studies and prioritize translationally relevant investigations [220]. Key priorities include systematic physicochemical characterization, quantitative pharmacokinetic and biodistribution analyses, long-term safety evaluation, clinically relevant tumor models, and direct comparisons with existing standard-of-care therapies [217]. Particular attention should be devoted to understanding how dynamic chemical transformations, tumor heterogeneity, and microenvironmental factors influence therapeutic outcomes. The integration of diagnostic and therapeutic functions may further enable mineral-based systems to sense and adapt to heterogeneous tumor environments, thereby improving treatment precision [221].

Ultimately, the future development of mineral-based anticancer therapeutics may benefit from a closed-loop “Composition–Mechanism–Delivery” design paradigm [222]. Within this framework, identification of active mineral components, elucidation of molecular mechanisms, and rational design of delivery systems are pursued simultaneously under rigorous safety-by-design principles [214]. Rather than treating formulation development as a downstream optimization step, delivery strategies should be designed to reinforce the intended therapeutic mechanism through tumor targeting, controlled ion release, organelle localization, redox modulation, or combination therapy [223]. Such an integrated approach may transform mineral drugs from empirically used materials into mechanistically defined and clinically translatable precision therapeutics, thereby unlocking the full potential of mineral resources in modern oncology [224].

## Figures and Tables

**Table 1:** Representative traditional mineral drugs and mineral-derived systems for cancer-related applications.

Category	Agent/System	Components	Applications	Status	Refs.
Direct anticancer	ATO	As_2_O_3_	APL and selected solid tumors	Clinical applications	[19,21]
Direct anticancer	RIF	As_4_S_4_-based formula	APL	Phase III clinical trials/clinical use in China	[22,23]
Direct anticancer	Realgar/nano-realgar	As_4_S_4_	Leukemia and selected solid tumors	Preclinical studies	[24,25]
Direct anticancer	Cinnabar-/HgS-containing mineral drugs	HgS	Tumor-like masses	Preclinical studies	[26,27]
Direct anticancer	Borax and boric acid derivatives	Na_2_B_4_O_7_·10H_2_O/boric acid	Hepatocellular carcinoma, glioblastoma, and small-cell lung cancer	Preclinical studies	[28,29]
Direct anticancer	Selenium-containing agents	Se/sodium selenite/selenium-based nanosystems	Chemoprevention, radiosensitization, and anticancer	Phase I clinical trials/preclinical studies	[30,31]
Direct anticancer/delivery platform	Iron-containing mineral drugs/SPIONs	Fe_3_O_4_/γ-Fe_2_O_3_	Magnetic hyperthermia, drug delivery, and ferroptosis-oriented tumor inhibition	Preclinical studies	[32,33]
Antitumor/adjunctive care	Mirabilite (Mangxiao)	Na_2_SO_4_·10H_2_O	Malignant pleural effusion and colorectal cancer	Clinical supportive use/preclinical studies	[34,35]
Adjunctive care	Gypsum/calcium sulfate	CaSO_4_·2H_2_O	Cancer-related fever and postoperative tissue	Clinical supportive use	[36,37]
Adjunctive care	Magnesium sulfate (Epsom salt)	MgSO_4_	Postoperative gastrointestinal recovery	Clinical supportive use	[38,39]
Local delivery	Hydroxyapatite- and calcium salt carriers	Hydroxyapatite/calcium sulfate-based biomaterials	Bone tumor-related reconstruction	Preclinical studies	[40,41]

**Table 2:** Quantitative toxicological comparison of conventional, processed, and nanoformulated mineral drugs.

Comparison Pair	Quantitative Toxicity Comparison	Main Toxicological Concern	Key Safety Interpretation	Refs.
ATO vs. realgar	ATO: oral LD_50_ of As_2_O_3_ is 33–39 mg/kg in mice. Realgar: oral LD_50_ approximately 3.2 g/kg.	ATO: heart block, hepatotoxicity, encephalopathy, carcinogenicity, and embryo-fetal toxicity. Realgar: hepatic and renal toxicity.	Poorly soluble arsenic sulfides and impurity removal lower acute systemic arsenic exposure. Realgar safety depends on the speciation, soluble content, purity, dose, and duration.	[51]
Copper ions vs. copper nanoparticles	Copper ions: reference. Copper nanoparticles: LD_50_ increased approximately 2.8-fold for 30 nm, 4.9-fold for 50 nm, 5.8-fold for 80 nm, and >13.9-fold for 1 μm.	Liver toxicity and kidney toxicity.	Larger particle size and controlled copper release may reduce acute toxicity, but organ accumulation and chronic toxicity remain key translational concerns.	[155]
Copper ions vs. copper oxides	Copper ions: oral LD_50_ value used as reference. Copper oxides: oral LD_50_ values are >14-fold higher and >13.9- fold higher.	Gastrointestinal, hepatic, renal, and oxidative stress-related toxicities.	Poor solubility, form, and particle size reduce immediate systemic copper availability. Lower acute oral toxicity does not establish an anticancer therapeutic index.	[156]
Boric acid vs. borax	Boric acid: oral LD_50_ 3450 mg/kg (male rats) and 4080 mg/kg (female rats). Borax: LD_50_ 4550 mg/kg (male rats) and 4980 mg/kg (female rats); approximately 1.3-fold and 1.2-fold higher.	Testicular atrophy, reduced fertility, decreased fetal body weight, and skeletal abnormalities.	Both have low acute oral toxicity, yet reproductive/developmental toxicity limits safety; systemic absorption depends on exposure route and skin integrity.	[157]
Cinnabar/HgS-containing mineral drugs vs. soluble mercury species	NR	Mercury accumulation, renal dysfunction, neurotoxicity, and organ-specific injury.	Washing, levigation, and quality control must separate poorly soluble HgS from toxic soluble mercury or free contamination; translational feasibility then depends on stability, purity, and long-term accumulation.	[93]
Ferumoxytol/SPIONs formulation vs. non-clinical iron oxide systems	NR	Hypersensitivity/anaphylaxis, hypotension, iron overload, MRI interference.	Carbohydrate coating boosts stability and iron handling, but iron replacement does not prove oncology safety; tumor dosing requires separate validation.	[158]

**Table 3:** Quantitative efficacy, pharmacokinetic, and release-related comparison of representative mineral-based anticancer formulations.

Agent/Formulation	Reported Efficacy Data	Reported PK, Biodistribution, or Release Data	Calculated or Comparative Interpretation	Refs.
ATRA + ATO vs. ATRA + chemotherapy	ATRA-ATO: Complete remission. ATRA-chemotherapy: 2-year EFS was 97% vs. 86%.	2-year EFS: 11 percentage points higher than that with ATRA + chemotherapy.	ATRA + ATO vs. ATRA + chemotherapy	[80]
ATRA + RIF vs. ATRA + intravenous ATO	ATRA-RIF: 2-year DFS was 97%. ATRA-ATO: 98%.	2-year DFS: 1 percentage point difference vs. intravenous ATO.	ATRA + RIF vs. ATRA + intravenous ATO	[51]
ATO-loaded liposomes vs. free ATO/control	ATO-loaded liposomes: 61.2% tumor inhibition (S180 mice); >80% entrapment; reduced plasma clearance; increased T_1/2_ and AUC0-12 h.	Improved tumor inhibition and systemic exposure profiles.	ATO-loaded liposomes vs. free ATO/control	[170]
ATO microcrystal-loaded PLGA microspheres vs. free ATO/previous ATO nano- or microparticles	ATO microcrystal-loaded PLGA microspheres: 80% tumor inhibition (HCC model), 40.1% drug-loading efficiency, 4–20-fold higher.	Drug loading: 4–20-fold higher than that of previous ATO nano-or microparticle systems.	ATO microcrystal-loaded PLGA microspheres vs. free ATO/previous ATO nano- or microparticles	[171]
Nano-realgar hydrogel/NRA@DH Gel + RT vs. NRAQDs, RT alone, gel control, or saline	NRA@DH Gel + RT: 0.30 (TAR), tumor CI: 5.88 and 3.82 on days 2 and 3, pH-sensitive hydrogel enabled sustained release. Saline control group: 14.38 (TAR), RT-alone: 1.09 (TAR).	TAR: 47.9-fold Lower than that of saline control and 3.6-fold lower than that of RT alone.	Nano-realgar hydrogel/NRA@DH Gel + RT vs. NRAQDs, RT alone, gel control, or saline	[172]
Cr-loaded CuO NPs vs. free Cr or CuO NPs	Cr@CuO NPs: IC_50_ 5.17 ± 0.48 μg/mL, entrapment 78.9 ± 5.9%, pH 5.5 release: 26.42% (2 h), 77.30% (12 h). Free Cr: 8.24 + 0.74 ug/mL, CuO NPs: 11.25 ± 1.04 μg/mL.	Cr@CuO NPs: IC_50_ reduced by approximately 37.3% vs. free Cr and by 54.0% vs. CuO NPs alone.	Cr-loaded CuO NPs vs. free Cr or CuO NPs	[173]
CuO NPs/5-FU-loaded Cu gel vs. free 5-FU or CuO NPs	PXFCu6 gel: IC_50_ 11.82 ± 0.22 μg/mL, controlled/sustained release: >8 h. Free 5-FU: 19.3 ± 0.49 μg/mL, CuONPs: 42.8 ± 0.24 μg/mL.	PXFCu6 gel: IC_50_ reduced by approximately 38.8% vs. free 5-FU and by 72.4% vs. CuO NPs alone.	CuO NPs/5-FU-loaded Cu gel vs. free 5-FU or CuO NPs	[174]
Se-MOP vs. control/other cancer cell lines	Se-MOP: IC_50_ 2 μg/mL at 48 h in HepG2 cells. Control/other cancer cell lines: NR.	NR.	Se-MOP vs. control/other cancer cell lines	[175]
Fu/NCur/SeNPs vs. cisplatin or individual components	Fu/NCur/SeNPs: IC_50_ 10.35 ± 0.83 mg/L (CaCo_2_ cells), 19.44 ± 1.39 mg/L (HT-29 cells). Cisplatin or individual components: NR.	NR.	Fu/NCur/SeNPs vs. cisplatin or individual components.	[176]

**Table 4:** Representative smart delivery strategies for mineral-based anticancer therapeutics.

Comparison Pair	Delivery Strategy/Responsive Trigger	Reported Release PK, or Biodistribution Data	Mechanistic and Translational Note	Refs.
ATO-loaded liposomes vs. free ATO/control	Liposomal encapsulation using a Cu(OAc)_2_ gradient	ATO-loaded liposomes: entrapment > 80%; reduced clearance; increased T_1/2_ and AUC0-12 h; 61.2% tumor inhibition (S180 mice).	Encapsulation improves arsenic retention, tumor-specific PK, biodistribution, and toxicity.	[186]
Angiopep-2-modified calcium arsenite-loaded liposomes vs. non-targeted/non-responsive formulations	Ligand-modified liposomes with acid-responsive release	Angiopep-2-modified calcium arsenite-loaded liposomes: high loading/entrapment; pH-responsive arsenic release; enhanced anti-glioma activity.	Angiopep-2 enables BBB/glioma targeting via LRP; acid-responsive release activates arsenic. PK and distribution data need standardization.	[187]
ATO microcrystal-loaded PLGA microspheres vs. free ATO/previous ATO nano-or microparticles	Polymeric microspheres for locoregional delivery/chemoembolization	ATO microcrystal-loaded PLGA microspheres: 40.1% drug-loading (4–20-fold higher), 80% tumor inhibition (HCC model).	Local retention increases tumor arsenic exposure and oxidative stress-mediated suppression. More suitable for local or interventional delivery than systemic delivery.	[171]
As@ZIF-8 nanoparticles vs. neutral pH release condition	pH-responsive coordination polymer	As@ZIF-8 nanoparticles: loading: 74 μg, minimal release at neutral pH, substantial under acidic conditions.	Acidic TME triggers arsenic release and tumor-selective cytotoxicity.	[188]
Nano-realgar hydrogel/NRA@DH gel vs. NRAQDs, RT alone, gel control, or saline	Hydrogel-based local retention and pH-sensitive release	Nano-realgar hydrogel/NRA@DH gel: tumor CI: 5.88 and 3.82 (days 2 and 3), hydrogel sustained release.	Nano-realgar sustained local exposure, radiosensitization tumor suppression (local GBM models only; systemic applicability unclear).	[172]
Cr-loaded CuO NPs vs. free Cr or CuO NPs	pH-associated release from copper-based nanocarrier	Cr-loaded CuO NPs: Cr entrapment 78.9 ± 5.9%; pH 5.5 release: 26.42% (2 h), 77.30% (12 h), IC_50_ 5.17 ± 0.48 μg/mL. Free Cr: IC_50_ 8.24 ± 0.74 μg/mL. CuO NPs: IC_50_ 11.25 ± 1.04 μg/mL.	Acidic TME triggers release, tumor-selective cytotoxicity (preclinical).	[173]
CuO NPs/5-FU-loaded Cu gel vs. free 5-FU or CuO NPs	Sustained local gel delivery	CuO NPs/5-FU-loaded Cu gel: sustained release >8 h, IC_50_ 11.82 ± 0.22 μg/mL. Free 5-FU: 19.3 ± 0.49 μg/mL, CuO NPs: IC_50_ 42.8 ± 0.24 μg/mL.	Sustained release: local exposure/potency (limited to in vitro/local delivery).	[174]
Enzyme-responsive mineral-based systems vs. non-enzyme-responsive systems	Enzyme-triggered release, such as MMP- or hyaluronidase-responsive designs	Enzyme-responsive mineral-based systems: mineral-drug release and distribution data limited.	Enzyme-rich TME: potential localized release (emerging; needs quantitative data)	[189]

## Data Availability

We are grateful for the mapping software by Figdraw (https://www.figdraw.com) for producing figures; no extra data was used for the research described in this article.

## Abbreviations

ALT	Alanine Aminotransferase
APL	Acute Promyelocytic Leukemia
APS	Advanced Planning and Scheduling
AST	Aspartate Aminotransferase
ATO	Arsenic Trioxide
ATRA	All-Trans Retinoic Acid
AUC	Area Under the Curve
CAM	Chorioallantoic Membrane
CHDT	Compound Huangdai Tablet
CI	Contrast Index
Cr	Platinum (Il)-based complex
CRC	Colorectal Cancer
DFS	Disease-free Survival
ECG	Electrocardiogram
EFS	Sevent-free Survival
EGCG-RNPs	(-)-Epigallocatechin-3-Gallate Encapsulated Realgar Nanoparticles
EZH2	Enhancer of Zeste Homolog 2
FoxO3a	Forkhead Box O3a
FXR	Farnesoid X Receptor
GPx	Glutathione Peroxidase
GPX4	Glutathione Peroxidase 4
GSH	Glutathione
GEM-MNP-pHLIP	G-PON Encapsulation Mode-Magnetic Nanoparticle-PH-Low Insertion Peptide
HCC	Hepatocellular Carcinoma
HgS	Mercuric Sulfide
IC_50_	Half Maximal Inhibitory Concentration
ICD	Immunogenic Cell Death
IFN-γ	Interferon-γ
IL-6	Interleukin-6
KM	Kunming
LD_50_	Median Lethal Dose
LRP	Lipoprotein Receptor-related Protein
MAPK	Mitogen-activated Protein Kinase
MDM2	Mouse Double Minute 2 Homolog
MMP-2	Matrix Metalloproteinase-2
MPE	Malignant Pleural Effusion
mPTP	Mitochondrial Permeability Transition Pore
MRI	Magnetic Resonance Imaging
N/A	Not Applicable
NDs	Nanodiamonds
NR	Nanodiamonds Not Reported
NRA	Nano-realgar
PEG	Polyethylene glycol
PK	Pharmacokinetics
PLGA	Poly (lactic-co-glycolic acid)
PML	Promyelocytic Leukemia
rhG-CSF	Recombinant Human Granulocyte Colony-Stimulating Factor
RT	Radiotherapy
RTS	Realgar-containing Serum
RIF	Realgar-Indigo Naturalis Formula
ROS	Reactive Oxygen Species
SeNPs	Selenium Nanoparticles
SPIONs	Superparamagnetic Iron Oxide Nanoparticles
T_1/2_	Half-life
TAR	Tumor Area Ratio
TCM	Traditional Chinese Medicine
TME	Tumor Microenvironment
TNF-α	Tumor Necrosis Factor-α
TSIIA@SeNPs-APS	TCM active ingredient-based SeNP surface decorated with APS and loaded with TSIIA
WHO	World Health Organization
ZIF-8	Zeolitic Imidazolate Framework-8
5-FU	5-fluorouracil
